# Supplementary figures and images for: Shedding Light on the Grey Zone of Speciation along a Continuum of Genomic Divergence
Source: PLoS Biol. 2016 Dec 27;14(12):e2000234. doi: 10.1371/journal.pbio.2000234 (PMC5189939; doi:10.1371/journal.pbio.2000234)

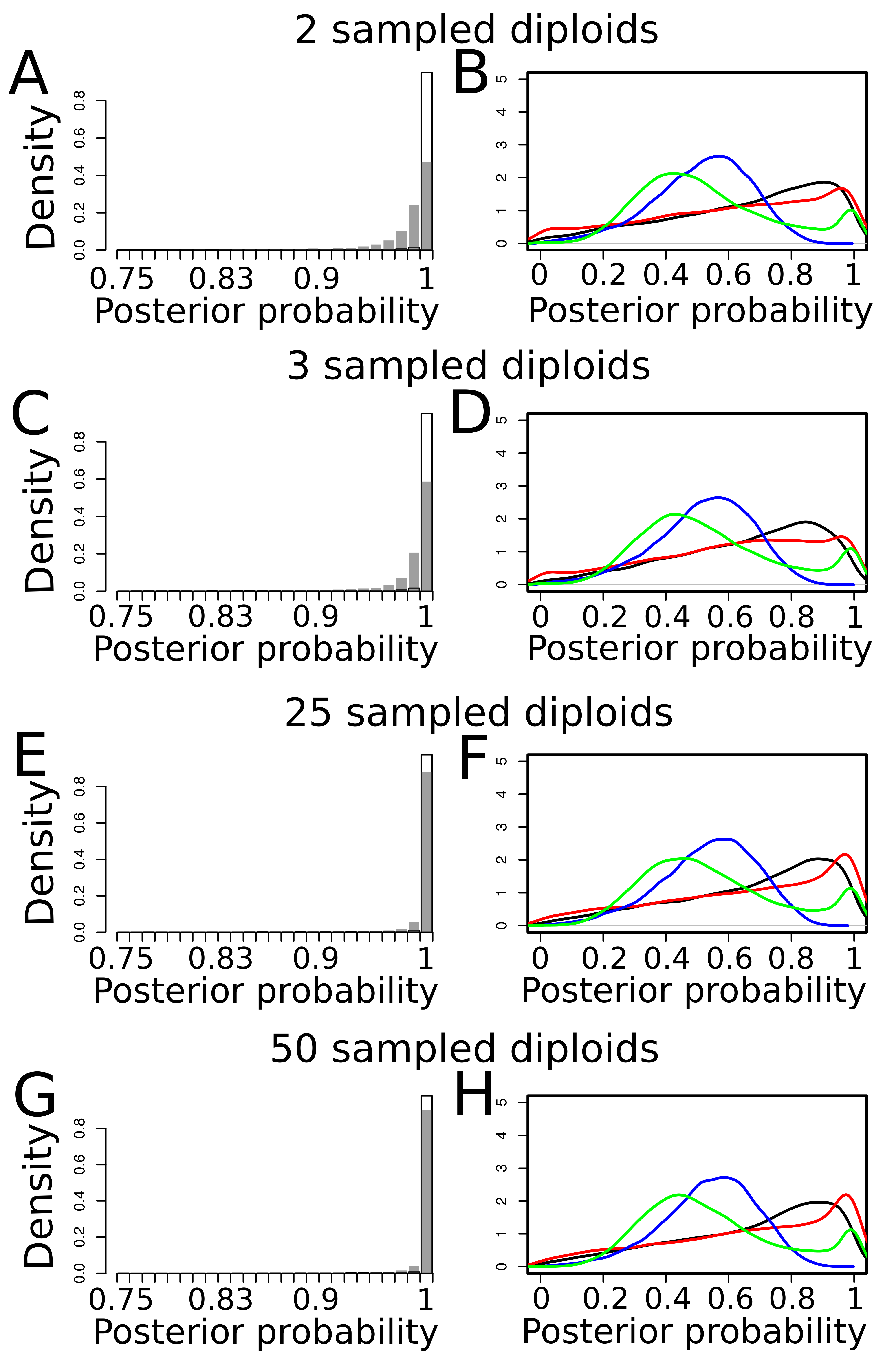

Supplement: S1 Fig — Analyses were made by simulating four different datasets: A-B: 100 loci sampled in two diploid individuals in each daughter species.C-D: 100 loci sampled in three diploid individuals in each daughter species.E-F: 100 loci sampled in 25 diploid individuals in each daughter species.G-H: 100 loci sampled in 50 diploid individuals in each daughter species. Panels on the left border show the distributions of P(current isolation | current isolation) (white bars) and P(current introgression | current introgression) (grey bars) measured after ABC analysis of 20,000 PODs simulated under each models. Panels on the right border show the distributions of P(SI | SI) (black lines), P(AM | AM) (red lines), P(IM | IM) (blue lines) and P(SC | SC) (green bars) measured after ABC analysis of 20,000 PODs simulated under each models. (TIF) [file pbio.2000234.s001.tif]

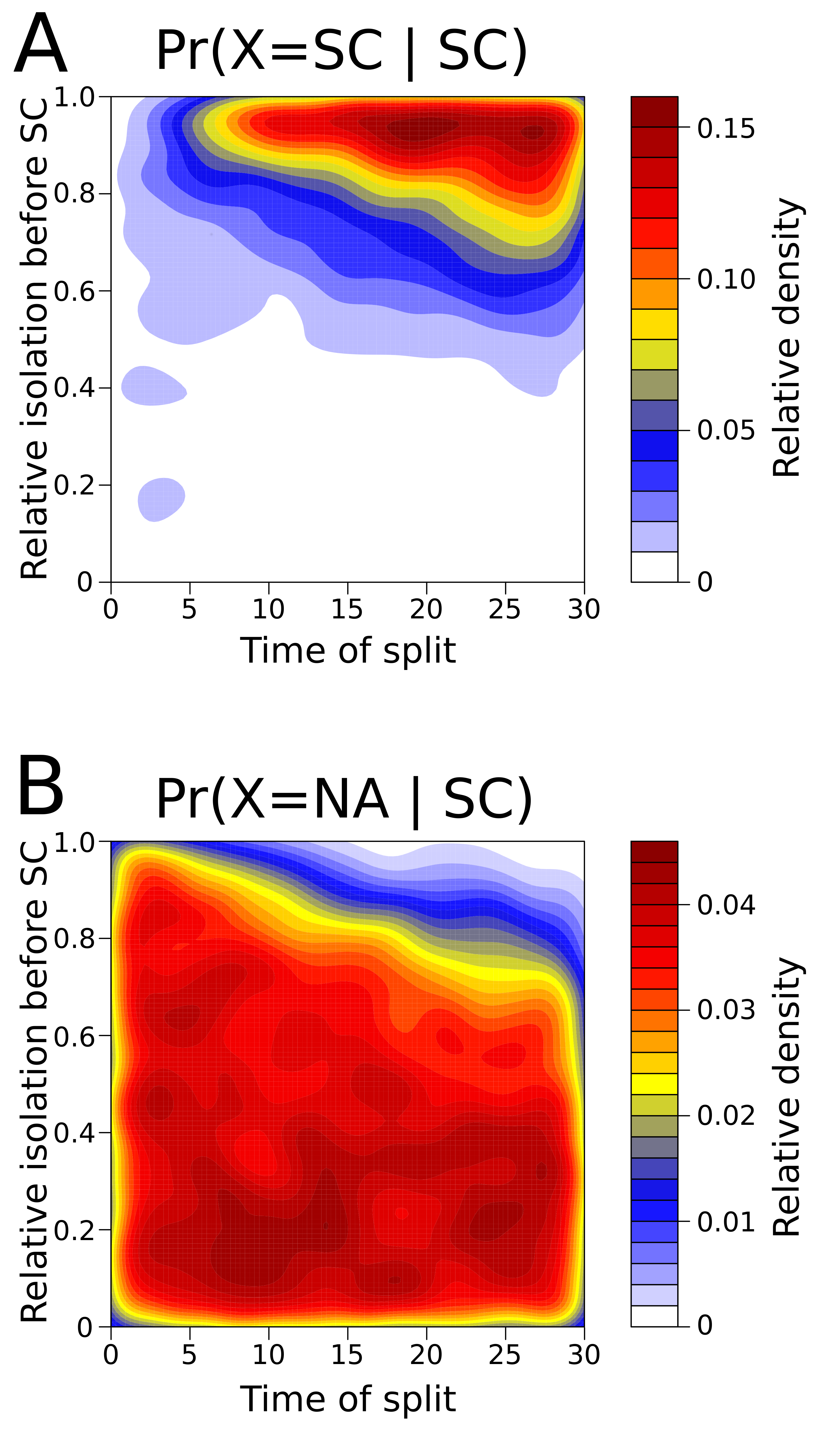

Supplement: S2 Fig — A. Two-dimensional space of parameters of the SC model showing simulations leading to a correct support of SC (i.e P(SC | SC) > 0.8). X-axis represents the time since the ancestral split. Y-axis represents the relative time the two daughter species remained isolated before the secondary contact. Colors represent the density in simulations with P(SC | SC) > 0.8. B. Two-dimensional space of parameters of the SC model showing simulations leading to the absence of a robust conclusion using ABC. Colors represent the density in simulations with P(NA | SC). (TIF) [file pbio.2000234.s002.tif]

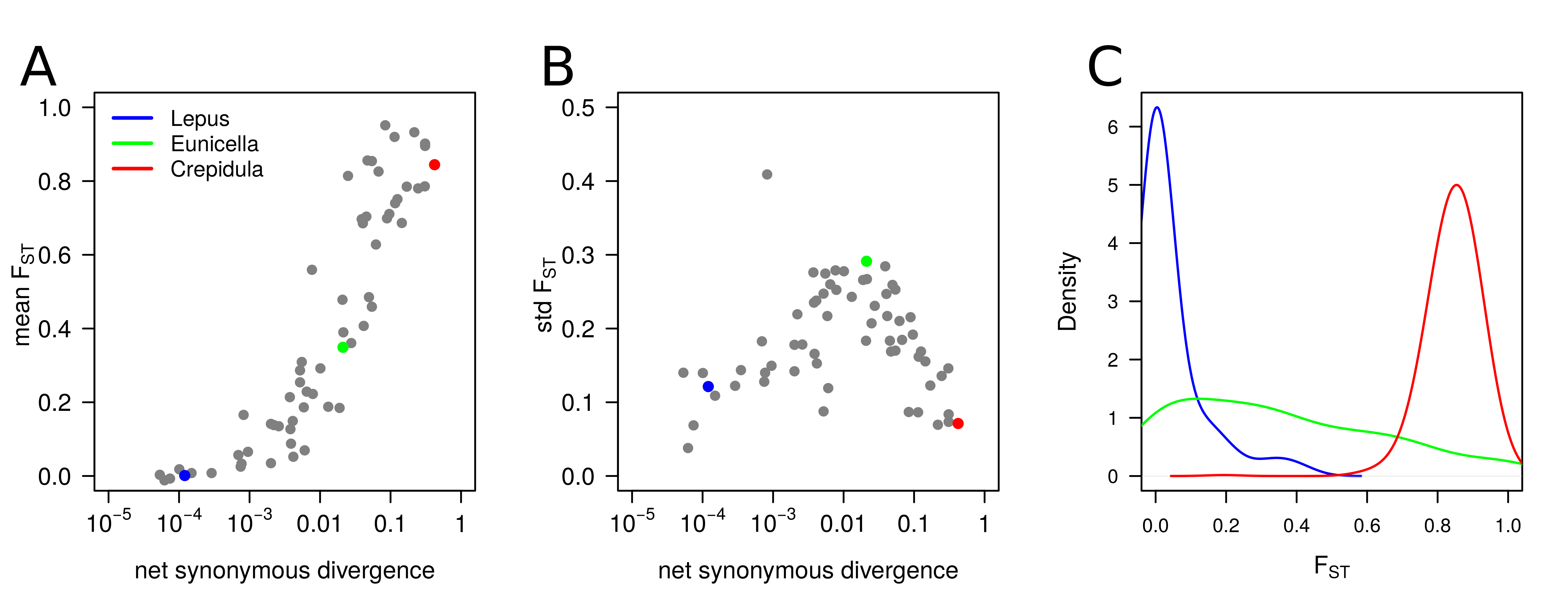

Supplement: S3 Fig — Each grey dot represents a pair of species/populations. Lepus (Spanish and Portuguese populations of Lepus granatensis), Eunicella (Eunicella cavolinii and E. verrucosa) and Crepidula (Crepidula fornicata and Bostrycapulus aculeatus) indicate representative pairs of poorly, intermediately and highly divergent species/populations. Effect of divergence on across-loci variance in FST. Genomic distribution of FST for the Lepus, Eunicella and Crepidula datasets (see S1 Data). (TIF) [file pbio.2000234.s003.tif]

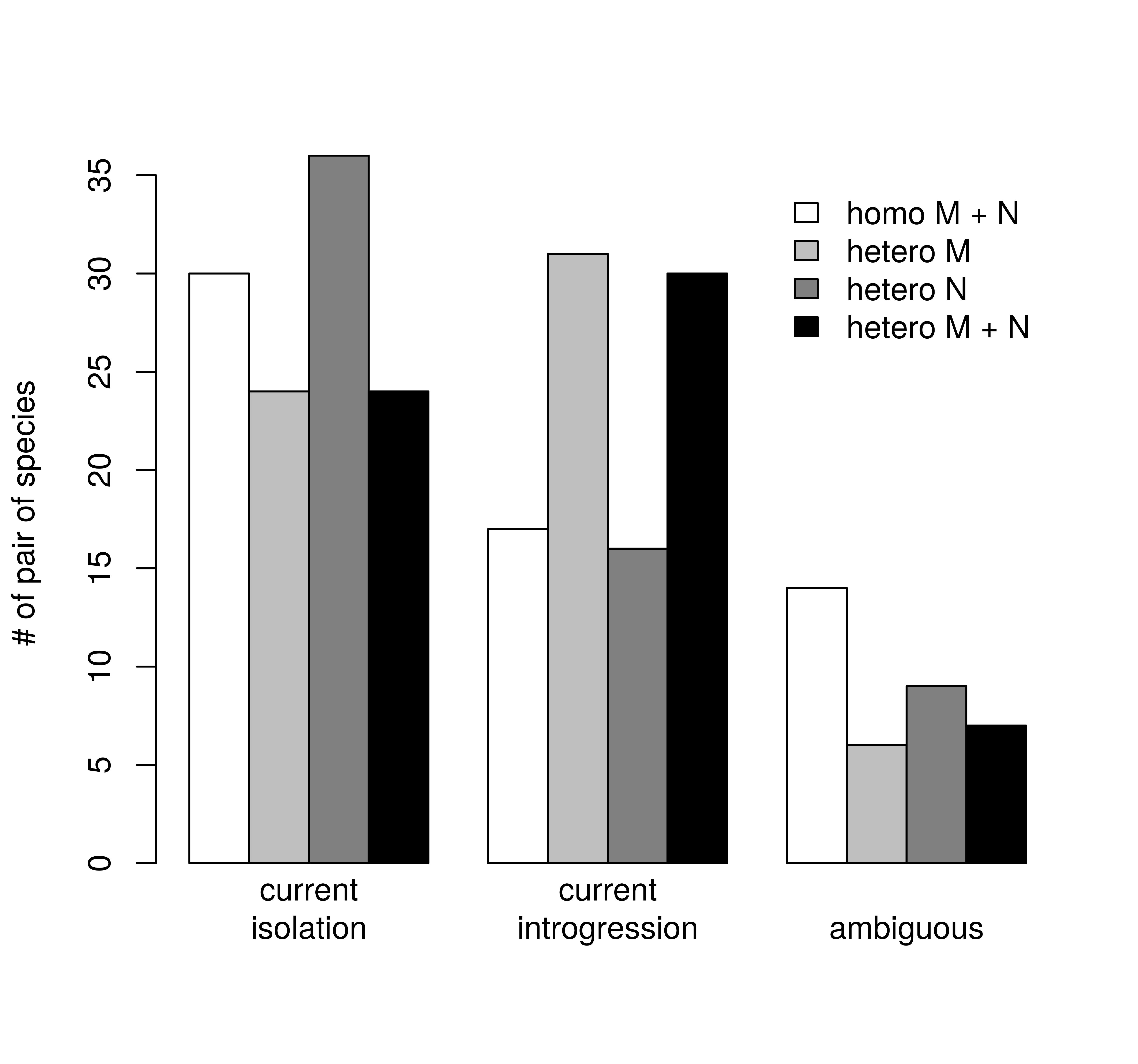

Supplement: S8 Fig — A pair of species is associated to “current isolation” if the sum of posterior probabilities P(SI) + P(AM) is associated to a robustness ≥ 0.95. A pair of species is associated to “current introgression” if the sum of posterior probabilities P(SC) + P(IM) is associated to a robustness ≥ 0.95. The ambiguous status is attributed to a pair of species when “current isolation” and “current introgression” are not strongly supported. The “homo M + N” analysis was made by assuming an unique genomic introgression rate and an unique Ne over the whole genome. The “hetero M” analysis takes into account genomic variation in introgression rates over the whole genome. The “hetero N” analysis takes into account genomic variation in Ne. The “hetero M + N” analysis takes into account genomic variation in introgression rates and in Ne (see S1 Data). (TIF) [file pbio.2000234.s008.tif]

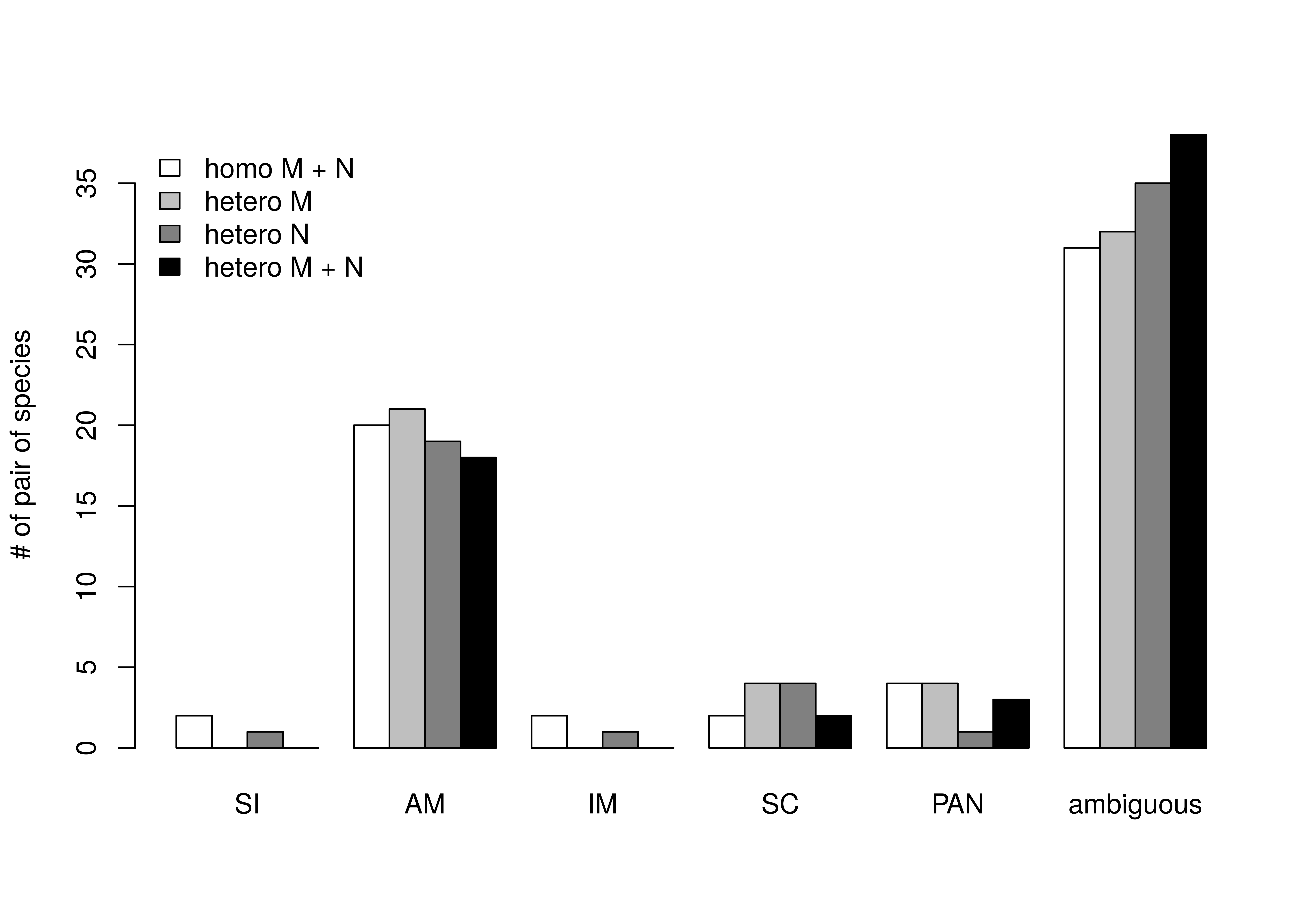

Supplement: S9 Fig — A pair of species is associated to SI or AM if its relative posterior probability is greater than 0.8696. A pair of species is associated to IM, SC or PAN tf its relative posterior probability is greater than 0.6419. The “homo M + N” analysis was made by assuming an unique genomic introgression rate and an unique Ne over the whole genome. The “hetero M” analysis takes into account genomic variation in introgression rates over the whole genome. The “hetero N” analysis takes into account genomic variation in Ne. The “hetero M + N” analysis takes into account genomic variation in introgression rates and in Ne (see S1 Data). (TIF) [file pbio.2000234.s009.tif]

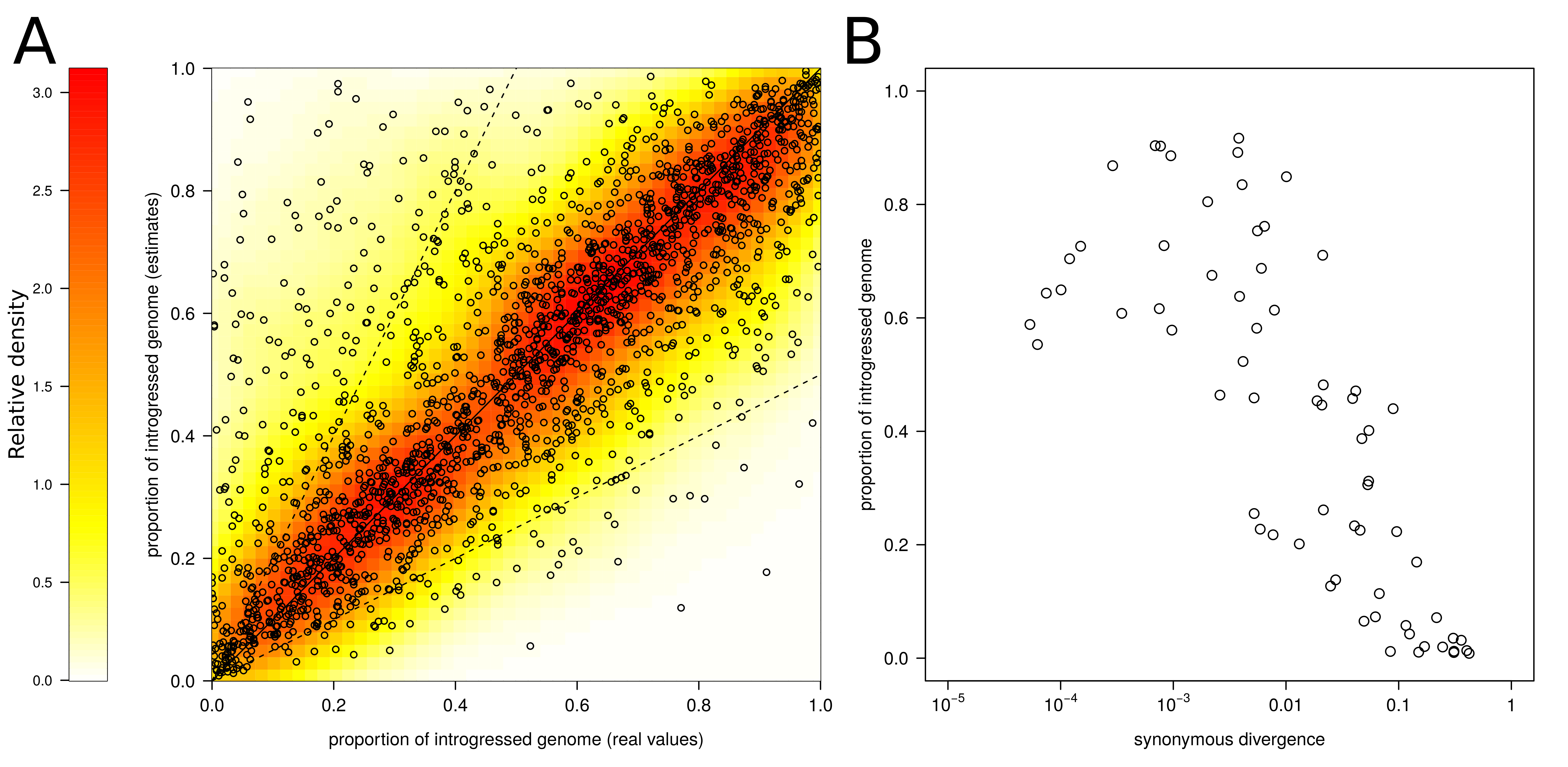

Supplement: S10 Fig — 2,000 pseudo-observed datasets (PODs) were simulated under the IM model with heterogeneity in introgression rates. We estimated the parameters of this model by using the ABC approach described in the ‘Materials and Methods’ section. α is the proportion of the genome crossing the species barrier at a rate N.m > 0. x-axis: values of α used to produce the PODs; y-axis: values of α estimated by ABC from the simulated PODs. Solid line represents f(x) = x. Dotted lines represent f(x) = 2.x and f(x) = x/2 respectively. Estimated values of α for the observed pairs of population/species as a function of their net synonymous divergence. (TIF) [file pbio.2000234.s010.tif]

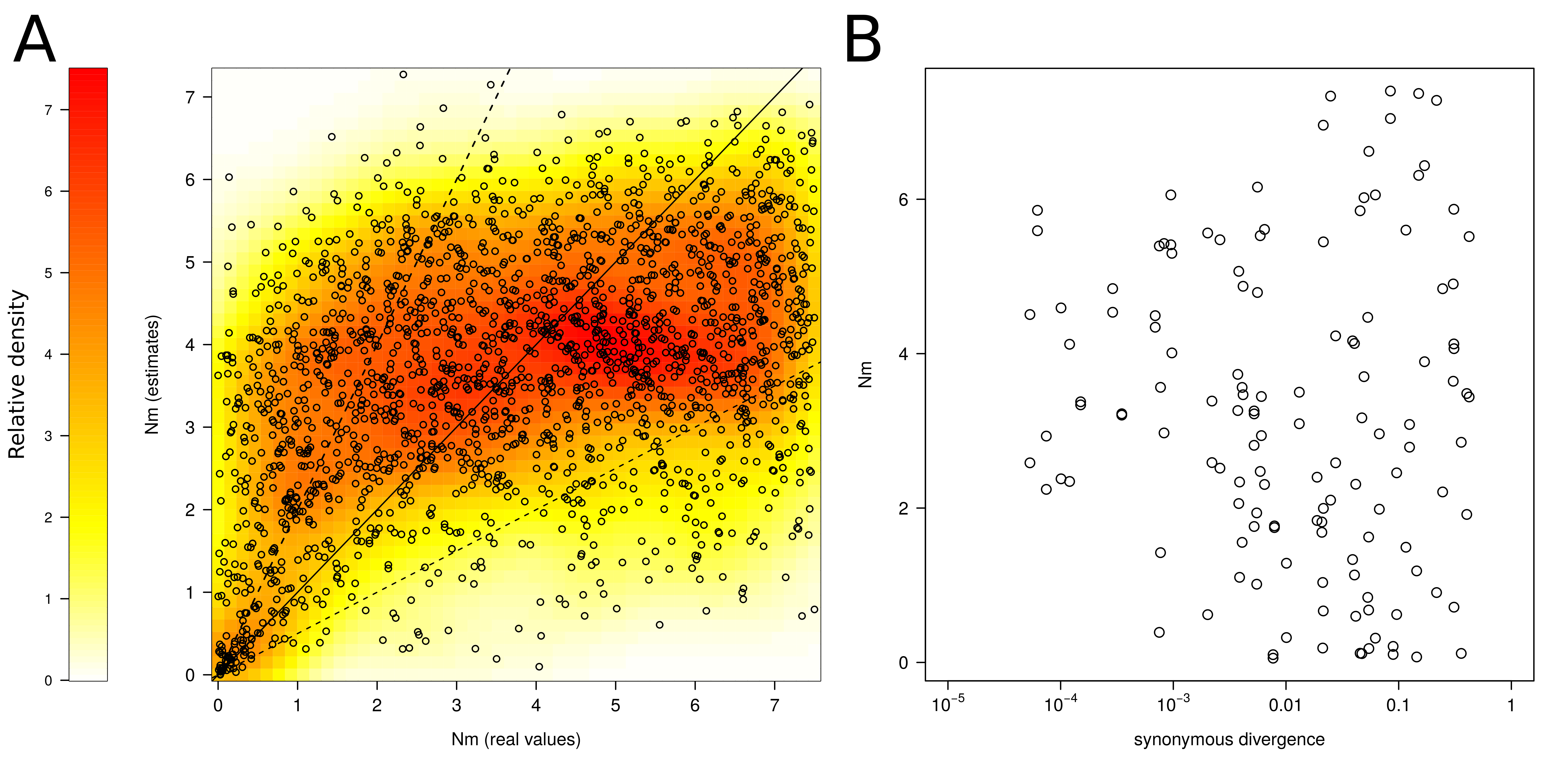

Supplement: S11 Fig — 2,000 pseudo-observed datasets (PODs) were simulated under the IM model with heterogeneity in introgression rates. A. x-axis: values of N.m used to produce the PODs; y-axis: values of N.m estimated by ABC from the simulated PODs. Solid line represents f(x) = x. Dotted lines represent f(x) = 2.x and f(x) = x/2 respectively. B. Estimated values of N.m for the observed pairs of population/species as a function of their net synonymous divergence. (TIF) [file pbio.2000234.s011.tif]

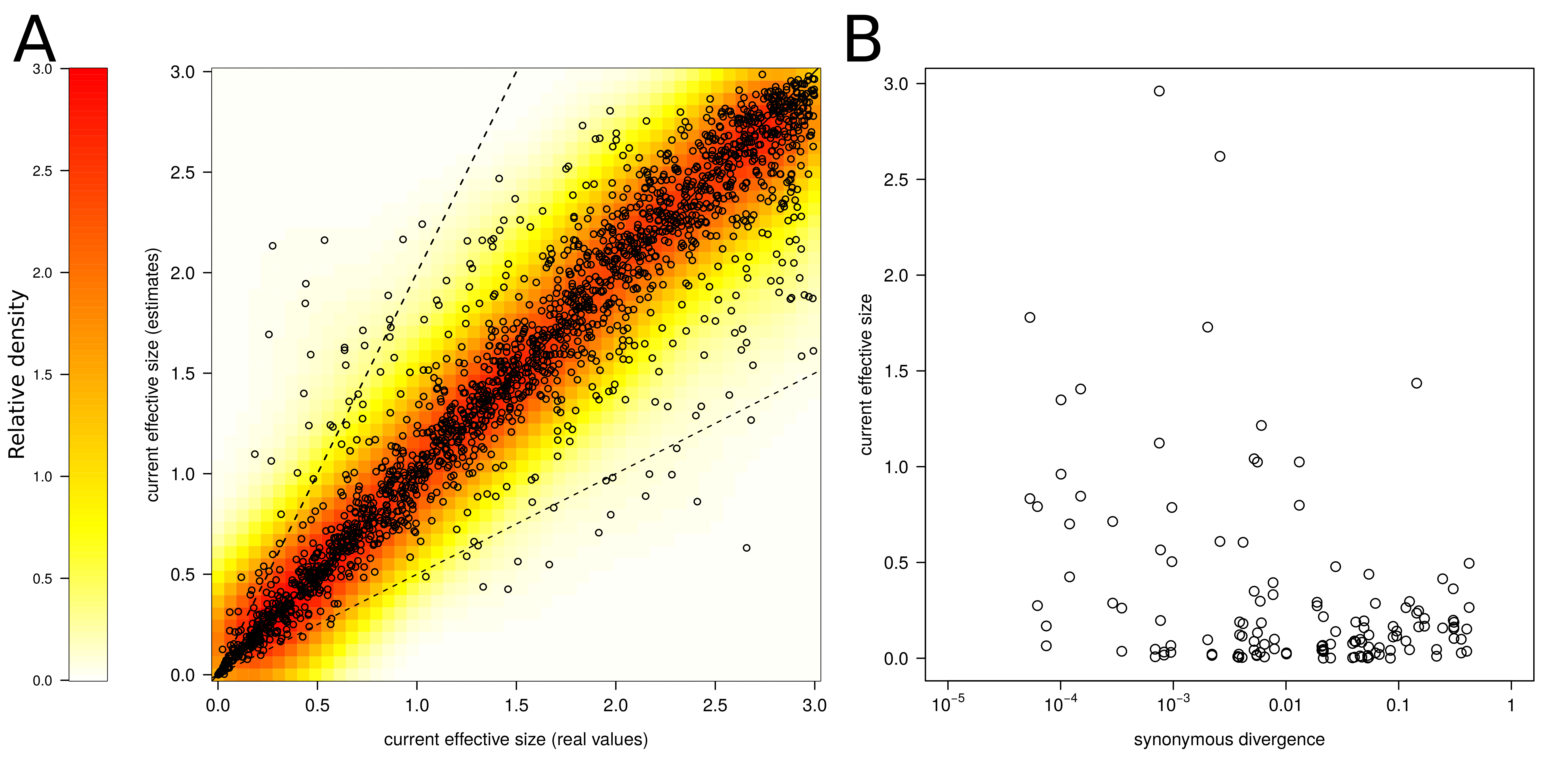

Supplement: S12 Fig — 2,000 pseudo-observed datasets (PODs) were simulated under the IM model with heterogeneity in introgression rates. A. x-axis: values of N used to produce the PODs; y-axis: current values of N estimated by ABC for all PODs. Solid line represents f(x) = x. Dotted lines represent f(x) = 2.x and f(x) = x/2 respectively. B. Estimated values of N for the observed pairs of population/species as a function of their net synonymous divergence. (TIF) [file pbio.2000234.s012.tif]

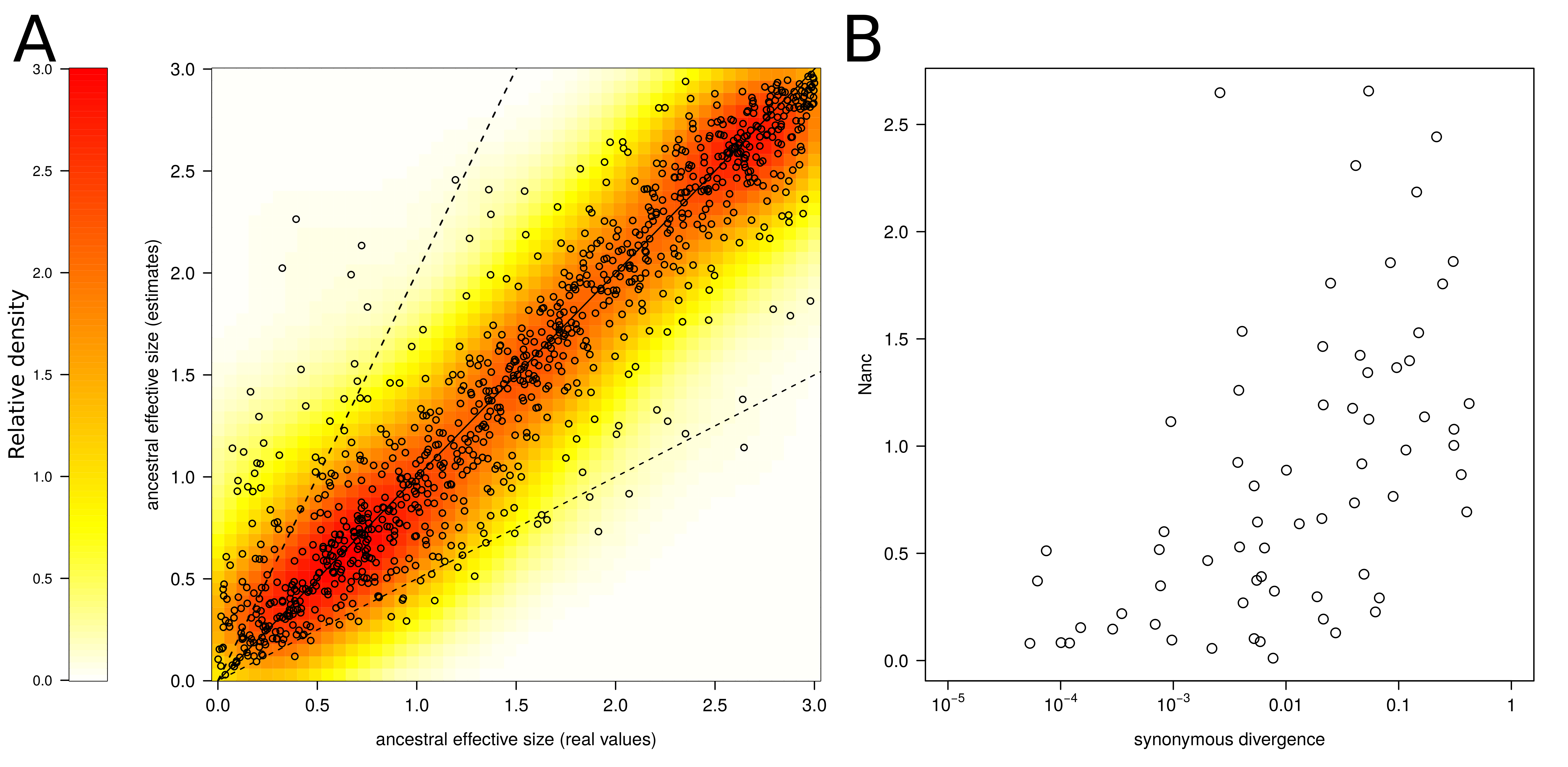

Supplement: S13 Fig — 2,000 pseudo-observed datasets (PODs) were simulated under the IM model with heterogeneity in introgression rates. A. x-axis: values of Nanc used to produce the PODs; y-axis: estimated values of Nanc for all PODs. Solid line represents f(x) = x. Dotted lines represent f(x) = 2.x and f(x) = x/2 respectively. B. Estimated values of Nanc for the observed pairs of population/species as a function of their net synonymous divergence. (TIF) [file pbio.2000234.s013.tif]

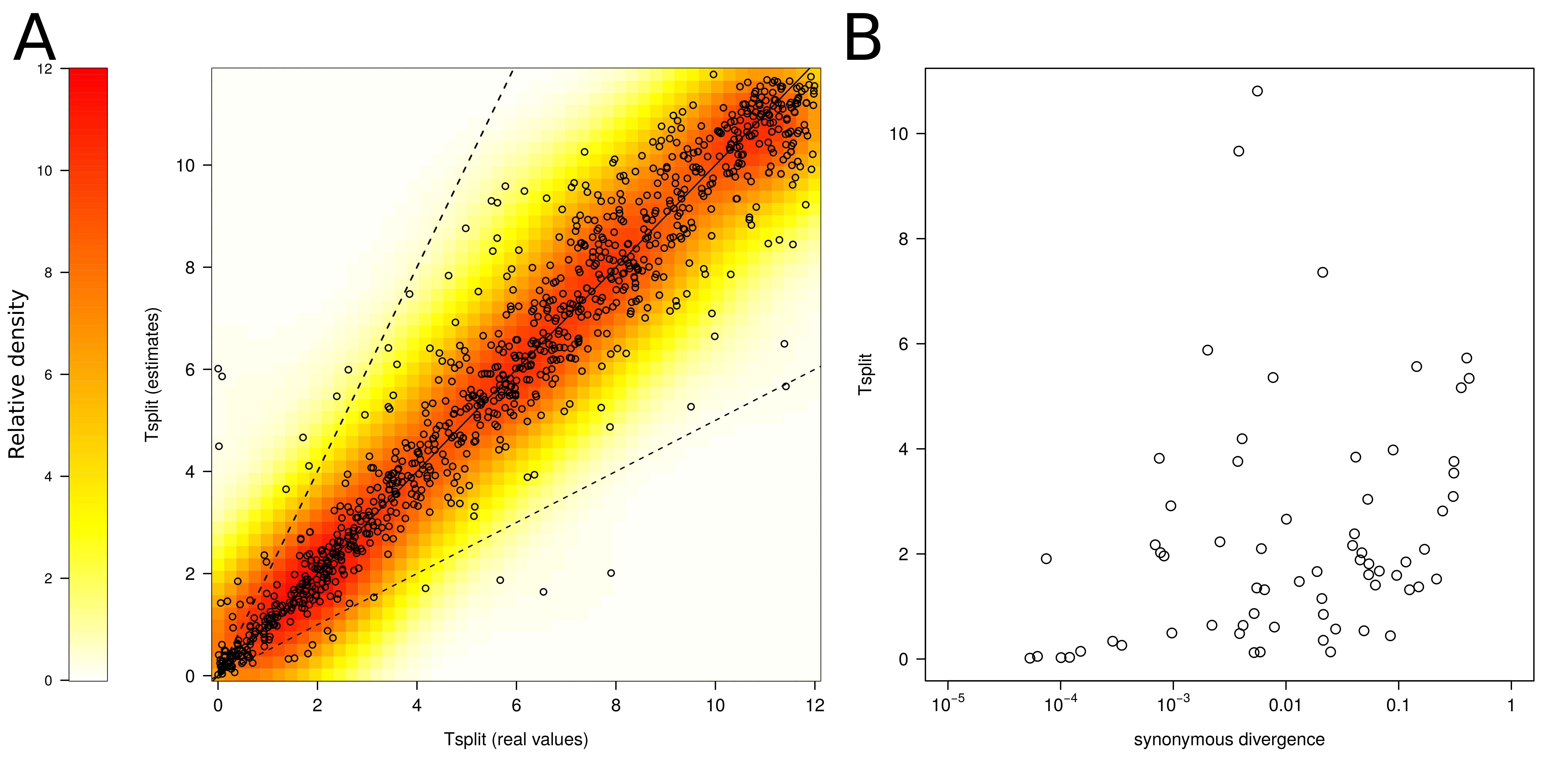

Supplement: S14 Fig — 2,000 pseudo-observed datasets (PODs) were simulated under the IM model with heterogeneity in introgression rates. Tsplit is expressed in million of generations since the ancestral separation. A. x-axis: values of Tsplit used to produce the PODs; y-axis: estimated values of Tsplit for all PODs. Solid line represents f(x) = x. Dotted lines represent f(x) = 2.x and f(x) = x/2 respectively. B. Estimated values of Tsplit for the observed pairs of population/species as a function of their net synonymous divergence. (TIF) [file pbio.2000234.s014.tif]
