# Supplementary material for: Shedding Light on the Grey Zone of Speciation along a Continuum of Genomic Divergence
Source: PLoS Biol. 2016 Dec 27;14(12):e2000234. doi: 10.1371/journal.pbio.2000234 (PMC5189939; doi:10.1371/journal.pbio.2000234)
Supplement: S1 Text — (PDF) [file pbio.2000234.s016.pdf]

Assessing the biological relevance of ABC along a continuum of divergence

Migration between two related gene pools is a major component of demographic history shaping patterns of polymorphism and divergence. However, the lack of direct observation of past demographic events makes their detection from current genomic data a central challenge in evolutionary biology. Recently, two simulation studies [1-2] highlighed the difficulty to correctly reject the SI model when the time of speciation is small compared to effective population sizes (ref and ref). More precisely, Hey et al. (2015) [1] analyzed 100 pseudo-observed datasets (PODs) simulated under the SI model with $\theta = 4Nu = 5$ and a $T_{split} = 0.5$, and found little signal to discriminate between SI and IM.

Here we assess how ABC discriminate between SI and IM when T_{split} tends to zero by using a simulation-based methodology similar to [1]. We reproduce below the pseudo-code describing the analysis.

.Loop over 100 values of T_{split} in {0.01, 0.02, … , 1}; θ=5 :
    .Loop over 100 replicated inferences for a given T_{split} value :
        .Random simulation of the multilocus pseudo-observed dataset POD_i under the SI model.
        .Estimation of the relative posterior probabilities pSI_i and pIM_i for the simulated POD_i.
        .If $pSI_i > pIM_i$:
            .robustness = $P(pSI_i \mid SI) / [P(pSI_i \mid SI) + P(pSI_i \mid IM)]$
            .if robustness ≥ 0.95 :
                True discovery of SI.
            .else :
                Statistical support is considered as ambiguous.
        .If $pIM_i > pSI_i$:
            .robustness = $P(pIM_i \mid IM) / [P(pIM_i \mid IM) + P(pIM_i \mid SI)]$
            .If robustness ≥ 0.95 :
                False discovery of IM.
            .else :
                Statistical support is considered as ambiguous.

For any given value of T_{split} we estimated the true positive rate (proportion of PODs supporting SI with a robustness ≥ 0.95), the false positive rate (proportion of PODs supporting IM with a robustness ≥ 0.95), and the rate ambiguity (proportion of PODs with a robustness < 0.95). Our results show that along a continuum of low divergence (Figure 1), the false positive rate varies between 0% and 4%. However, for very closely related pairs of populations ($T_{split} < 0.2$), the true positive rate is also very low, varying between 0% and 11%. Thus, for datasets simulated under the SI model with $T_{split} < 0.2$ (and $\theta = 5$), ABC rarely provides strong conclusion about the demographic model, with a rate of ambiguity lying from 89% to 100%. For $T_{split} > 0.2$, true positive rate increases, rate of ambiguity decreases and false positive rate remains at values below 4%. This analysis indicates that, although our ABC approach lose some power when T_{split} is very low, as expected, it is not affected by the bias of the IMa2 method documented by [1].

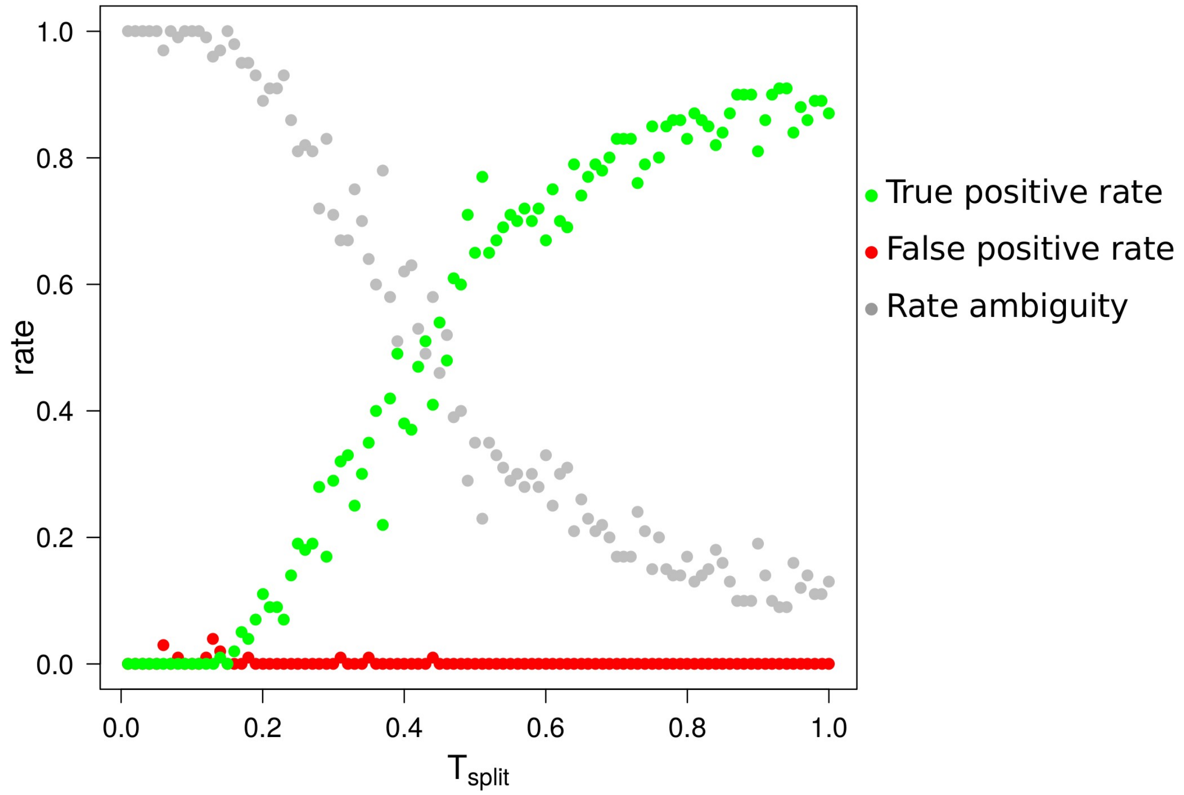

Figure 1: true positive, false positive and ambiguity rates along a continuum of low divergence: case of 2 models.

100 bins of T_{split} were explored along the x-axis. For each value, 100 PODs were simulated under the SI model with theta values fixed to 5.

Green dots: true positive rate ($P_{SI}>P_{IM}$ with robustness ≥ 0.95).

Red dots: false positive rate ($P_{SI}<P_{IM}$ with robustness ≥ 0.95).

Grey dots: rate ambiguity (robustness < 0.95).

The net synonymous divergence *Da* of the 10,000 simulated datasets lies from 0.0001 % to 0.2186 %, but the first true positive was found at *Da* = 0.032 % obtained when T_{split} = 0.14 (Figure 2).

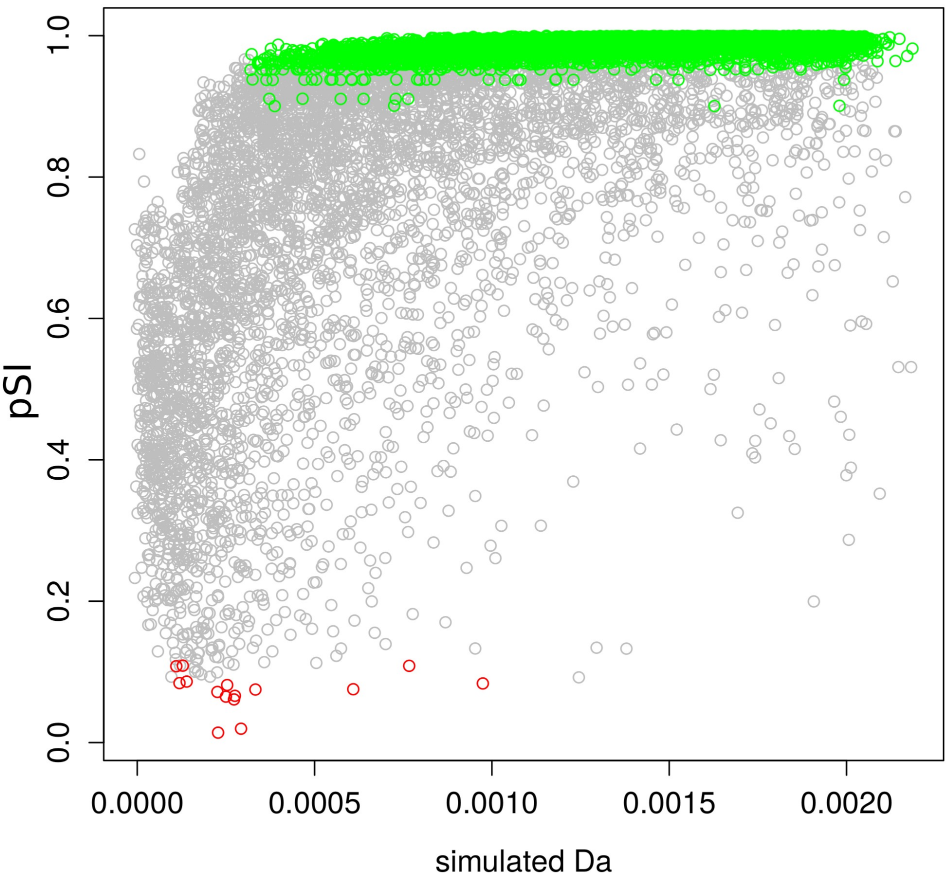

Figure 2: relative posterior probability pSI over 10,000 PODs simulated under the SI model as a function of the measured net divergence Da.

Green dots : PODs correctly supported as being SI with robustness ≥ 0.95.
Red dots : PODs wrongly supported as being IM with robustness ≥ 0.95.
Grey dots : ambiguous ABC inferences with robustness < 0.95.

We then estimated parameters of the IM model for each of the 10,000 PODs simulated under SI along a continuum of T_{split} and *Da* (Figure 3-A). There is no influence of Tspit on the accuracy of ABC to correctly estimate theta (Figure 3-B) and T_{split} (Figure 3-C). However, non-null migration rates are inferred when $T_{split} < 0.2$. Thus, investigators have to interpret cautiously ABC estimates of the IM parameters describing a very recent T_{split} with high gene flow since it can simply results from the SI model.

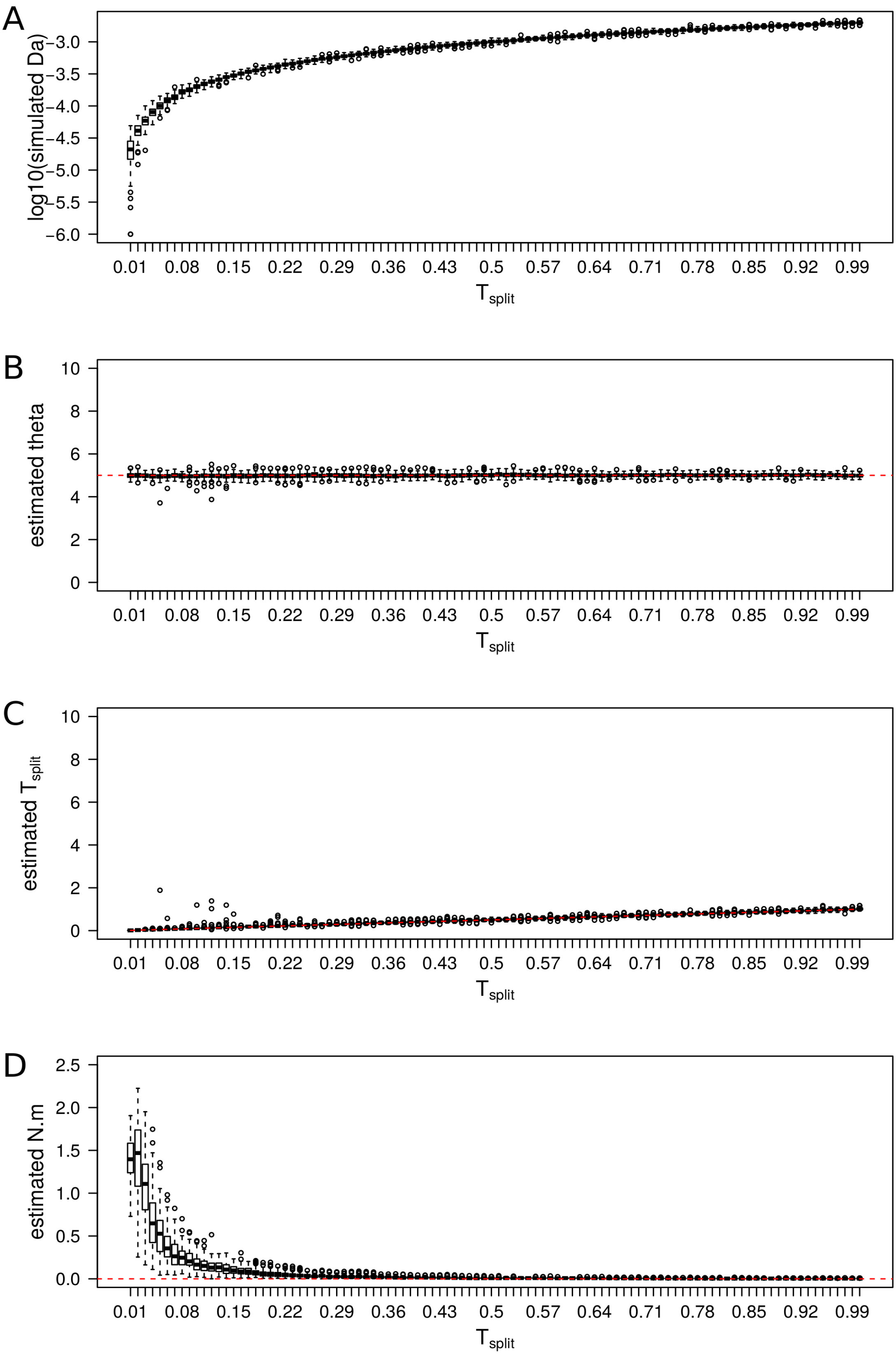

Figure 3: estimated parameters of the IM model for PODs simulated under SI .

Each boxplot represents observed values over 100 PODs simulated under the SI model with a fixed T_{split} value. A) Distribution of the average net divergence Da between simulated pairs of gene pools. B) Distribution of estimated θ by taking the median of posterior distributions of parameters. The red line shows the real value. C) Distribution of estimated Tsplit. D) Distribution of estimated migration rates. Y-axis enconcompasses the range of prior distributions.

Finally, we investigated more specifically the issues addressed by [1] to our system made by 16 alternative models. 116,000 PODs were generated by coalescent simulations under 16 models: 10 with ongoing gene flow (derivatives from IM, SC and PAN models) and 8 with current isolation (derivatives from SI and AM models). For each POD we estimated the posterior probability of "migration" vs "isolation" and tested the robustness of the best supported model. In this framework, we found that model comparison using ABC is a powerful and accurate approach to support ongoing migration (Figure 4-A) or current isolation (Figure 4-B) from data sets of the size of those analysed in this study. Besides its elevated true positive rates, the method has a low false positive rate, i.e., tends to return a message of ambiguity, rather than incorrect inference, when the signal is low (Figure 4-C; 4-D).

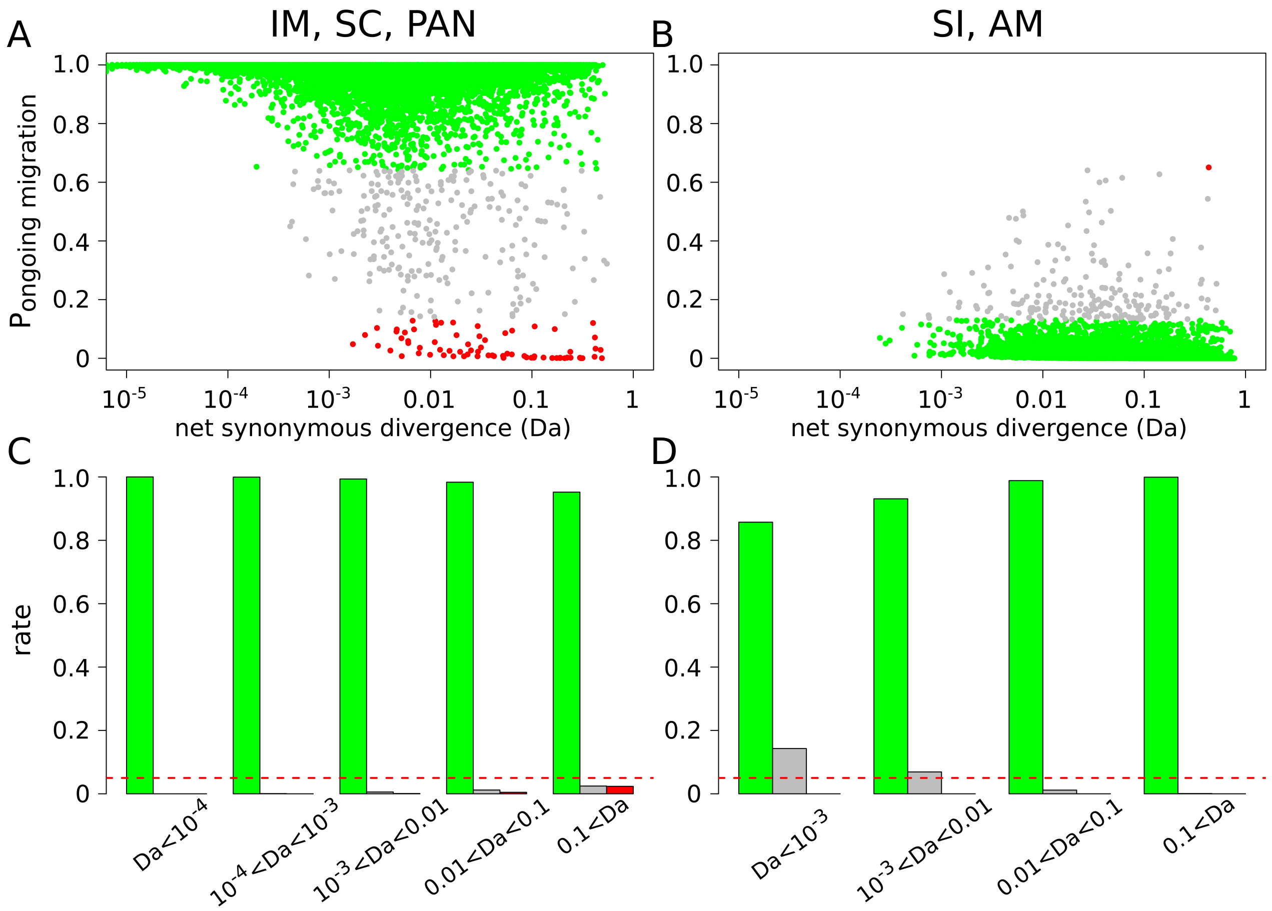

Figure 4: robustness of the ABC analysis along a continuum of divergence.

116,000 PODs were simulated under the 16 models surveyed in our study and analyzed under the same ABC protocol than for the 61 studied pairs of species.

A: probability to support ongoing migration as a function of *Da* when the good model assume ongoing migration (IM, SC or PAN). Dots show the results for each simulated pair of species. Green dots show the true positives (P_{GOOD}>P_{WRONG} with robustness ≥ 0.95), red dots show the false positives (P_{GOOD}<P_{WRONG} with robustness ≥ 0.95) and grey dots show ambiguous analysis (robustness < 0.95).

B: probability to support ongoing migration as a function of *Da* when the good model assume current isolation (SI or AM).

C: proportion of true positives, false positives and ambiguous analyses within different bins of divergence measure by *Da* when the good model assume ongoing migration.

D: proportion of true positives, false positives and ambiguous analyses within different bins of divergence measure by *Da* when the good model assume current isolation.

References

1. Hey J, Jody H, Yujin C, Arun S. On the occurrence of false positives in tests of migration under an isolation-with-migration model. Mol Ecol. 2015;24: 5078–5083.

2. Cruickshank TE, Hahn MW. Reanalysis suggests that genomic islands of speciation are due to reduced diversity, not reduced gene flow. Mol Ecol. 2014;23: 3133–3157.
